# Editorial: Wastewater-based epidemiological surveillance of respiratory pathogens

**DOI:** 10.3389/fpubh.2023.1328452

**Published:** 2023-11-16

**Authors:** David Champredon, Peter A. Vanrolleghem

**Affiliations:** ^1^Public Health Agency of Canada, Guelph, ON, Canada; ^2^Department of Civil Engineering and Water Engineering, modelEAU – Université Laval, Québec, QC, Canada

**Keywords:** wastewater, data quality, sampling methods, normalization, kinetics, epidemiology, respiratory infections

Monitoring the concentration of human pathogens in wastewater for public health has existed for over 80 years (1) but has rarely been performed for respiratory viruses until recently. Indeed, before the COVID-19 pandemic, there were few attempts by public health organizations to integrate the detection of respiratory pathogens in wastewater into their surveillance programs to monitor the prevalence of these infections in the population. This absence of enthusiasm may be explained by the unawareness that fecal (and urinary) shedding of respiratory viruses has a similar profile as the much-better-known respiratory shedding, making wastewater surveillance a potentially valuable indicator to monitor the dynamics of a respiratory epidemic. The COVID-19 pandemic has demonstrated the utility of wastewater-based epidemiology (WBE) during a major epidemiological event involving a respiratory pathogen and its potential value if integrated into existing epidemic surveillance programs.

This Research Topic showcases studies that highlight the emergence of the surveillance of respiratory pathogens from wastewater samples and also the potential challenges ahead.

Wastewater-based surveillance brings many potential benefits to public health activities, notably to complement (but not replace) other data sources as de Melo et al. show with student absenteeism data. One of the main goals of wastewater surveillance is to link the pathogen concentration in wastewater with hospitalizations associated with the pathogen. But, as Kadonsky et al. hint, the pathogen concentration in wastewater may be affected by factors unrelated to its epidemiology and perturb the wastewater concentration/hospitalization relationship. Normalization techniques (such as the one proposed by Dhiyebi et al.) are still being explored to account for exogenous effects to correct non-epidemiological effects on the wastewater signal.

Fecal shedding kinetics for an infected individual, a cornerstone of WBE, is poorly known for most pathogens. Even for the well-studied SARS-CoV-2, we don't know how (or even if) fecal shedding changed as the population got exposed to successive variants and different vaccines. In that spirit, Rioux et al. propose a clinical-data-driven method to correct initial assumptions about fecal kinetics.

Laboratory methods that accurately quantify viral concentration in wastewater are still in their early stages. We are probably several years away from a gold-standard laboratory method, assuming that “gold standard” even makes sense, given the diversity of the wastewater matrix across different sampling situations. In this Research Topic, the studies by Lucansky et al., Shinde et al., Nagelkerke et al., and Zhao et al. are moving research in that direction.

As an emerging field, WBE must find its place beside more established public health surveillance programs. There is probably no one-size-fits-all solution, but the study by Clark et al. can be helpful as they share a comprehensive framework that has been implemented in a large North American jurisdiction.

The COVID-19 pandemic showed the unexpected utility of WBE for respiratory pathogens. Leveraging this success, many jurisdictions are expanding their WBE to other respiratory pathogens, notably seasonal influenza (de Melo et al.) and RSV, historically the most burdensome respiratory diseases. Prioritizing and right-sizing WBE to new pathogens will be key to improving public health but may also be challenging given the specificities of each jurisdiction. To support such efforts, Gentry et al. propose a ranking system that was applied in a large North American urban center.

The choice regarding the geographical location of sampling sites can provide different spatial levels for epidemiological analyses. Sampling wastewater at wastewater treatment plants is a popular and practical choice to monitor the prevalence of a given infection at the (sub)municipal level. However, more focused sampling can bring unprecedented insights into fine-grained transmission patterns at key “hot spots”. For example, the study by Corchis-Scott et al. in this Research Topic samples university residences.

Clearly, WBE for respiratory pathogens is still in its infancy, and many knowledge gaps need to be filled. This Research Topic is a step in that direction. Given the recent expansion of WBE in many different jurisdictions worldwide, it is exciting to see research in this new field that promises to improve public health.

## Author contributions

DC: Writing—original draft, Writing—review & editing. PV: Writing—review & editing.
